# Microstructural Evolution of Post-Processed Hastelloy X Alloy Fabricated by Laser Powder Bed Fusion

**DOI:** 10.3390/ma12030486

**Published:** 2019-02-05

**Authors:** Giulio Marchese, Emilio Bassini, Alberta Aversa, Mariangela Lombardi, Daniele Ugues, Paolo Fino, Sara Biamino

**Affiliations:** Department of Applied Science and Technology, Politecnico di Torino, Corso Duca degli Abruzzi 24, 10129 Torino, Italy; emilio.bassini@polito.it (E.B.); alberta.aversa@polito.it (A.A.); mariangela.lombardi@polito.it (M.L.); daniele.ugues@polito.it (D.U.); paolo.fino@polito.it (P.F.); sara.biamino@polito.it (S.B.)

**Keywords:** laser powder bed fusion, additive manufacturing, Ni-based superalloys, hot isostatic pressing, microstructure characterization

## Abstract

Hastelloy X (HX) is a Ni-based superalloy which is employed to produce gas turbine and gas-cooled reactor sectors due to its outstanding oxidation resistance and high tensile strength at high temperatures. This alloy can be processed by laser powder bed fusion (LPBF) fabricating complex geometries in a single step. However, post-processing thermal treatments must be applied to generate a suitable microstructure for high-temperature applications. The investigation reports the microstructure evolution of LPBF HX samples under specific post-processing treatments. A hot isostatic pressing (HIP) treatment can close the internal cracks and reduce the residual porosity (less than 0.1%). Moreover, the HIP-triggered recrystallization generated equiaxed grains, while the slow cooling rate generated a film of intergranular carbides (Mo-rich M_6_C and Cr-rich M_23_C_6_) and intragranular carbides (Mo-rich M_6_C carbides). Therefore, a solution annealing was performed to dissolve the film of carbides which may reduce the ductility. The post solution annealed material consisted of equiaxed grains with ASTM grain size number mainly 4.5-5.5 and inter/intragranular Mo-rich M_6_C carbides. The microstructure is highly comparable with solution annealed wrought HX alloy. Finally, after simulating short thermal exposure at 745 °C for 6 h, a significant formation of Cr-rich M_23_C_6_ carbides was observed strengthening the LPBF HX alloy.

## 1. Introduction 

The recent development of additive manufacturing (AM) processes makes it possible to produce near-net-shape complex parts in a single step using a layer by layer process. These technologies are an enormous attraction for the production of components made of superalloys with very complex shapes, overcoming the issues related to their low machinability resulting from high hardness and strength [1,2,3]. 

Among the AM technologies, laser powder bed fusion (LPBF) is extensively employed to fabricate Ni-based superalloy components [4,5]. During the LPBF process, the laser beam melts very narrow areas of powder, generating very high heating and cooling rates (around 10^5^–10^6^ °C/s [2,4]), repeating it in consecutive layers in order to create the final components [6,7]. For Ni-based superalloys, the LPBF process involves the formation of extremely fine dendritic/cellular structures and inhibits the formation of large segregate areas. Moreover, the dissipation of heat flow from the melt pools to the substrate triggers the formation of columnar grains along the building direction leading to anisotropic mechanical properties [8,9,10,11]. 

The LPBF process has been successfully employed for the fabrication of dense components made of various Ni-based superalloys such as Inconel 625 and Inconel 718 [9,12,13]. However, several Ni-based superalloys are prone to cracking during the LPBF due to their low weldability or the inappropriate selection of process parameters [2,4,14,15,16,17]. For instance, Hastelloy X (HX) alloy is characterized by susceptibility to crack formation when processed by LPBF [2,18,19,20,21,22,23]. This alloy is a solid solution strengthened superalloy, which offers an exceptional oxidation resistance as well as high strength and ductility at high temperatures [18,24], making it the perfect candidate for the production of high-temperature components such as combustion parts in gas turbines as well as high-temperature gas cooled reactors [24,25,26].

Various phenomena have been proposed in the literature to explain the crack formation of LPBF HX alloy. Harrison et al. [2] found that a modified HX alloy with a higher concentration of solid solution strengthened elements (e.g., Cr, Mo, W) resulted in fewer cracks with respect to the standard HX alloy. They suggested that an added increment of the solid solution strengthened elements can increase the thermal shock resistance of the alloy, reducing the formation of cracks. On the other hand, Tomus et al. [20] produced crack-free HX samples using a reduced quantity of Mn and Si within the starting HX powder. They stated that the high concentration of minor elements such as Mn, Si, and C can enhance the sensitivity to crack formation due to the decreasing of the solidification temperature of the alloy and microsegregation at grain boundaries [21].

In a previous work of the authors on LPBF HX alloy, microstructural analyses revealed the presence of Mo-rich carbides close to the cracks along the grain boundaries, indicating that the cracks are attributed to intergranular carbides together with high residual stresses induced by the LPBF processes [27]. 

On the contrary, recently Sanchez-Mata et al. [23] reported the production of crack-free LPBF HX samples without altering the chemical composition of the HX alloy. However, the as-built HX state exhibits microstructure features not suitable for high-temperature applications, making it necessary to perform specific post-processing treatments and reduce the porosity level.

Hot isostatic pressing (HIP) can consolidate possible internal cracks and reduce the porosity, and also promote microstructure evolution [18,19,28,29,30]. According to the literature on LPBF HX alloy, the as-built and hot isostatic pressed (HIPed) HX samples revealed higher yield strength (YS) and ultimate tensile strength (UTS) but less ductility than hot forged HX alloy [18]. Moreover, Tomus et al. [30] studied the microstructures and mechanical properties of as-built and post-processed LPBF HX, performing a solution annealing, a HIP treatment, as well as a HIP treatment followed by solution annealing. The as-built and post-processed HX revealed higher YS and similar UTS with respect to the commercially available tensile data sheet on solution annealed wrought HX alloy, while the HIPed and post-solution annealed HX alloy exhibited higher ductility than solution annealed wrought HX alloy. Additionally, they showed the presence of very fine Mo-rich carbides along the grain boundaries of HX samples subjected to HIP and post-solution annealing treatments. However, the limited number of carbides can derive from the reduced quantity of carbon, five times inferior to the value reported in the ASTM B435 for the HX alloy. It is therefore possible to assume that HX alloy with a standard chemical composition results in the formation of more carbides thus strengthening the alloy. Han et al. [29] mainly focused their attention to the mechanical properties of as-built and HIPed HX alloy, revealing the increment of fatigue resistance for the HIPed state with respect to the as-built condition, due to the reduction in stress concentration and residual stresses. 

Nevertheless, little work has been done on the microstructure evolution of LPBF HX alloy under post-processing and simulating possible operative thermal exposures, taking into account the phases formed that could have a fundamental role in the mechanical properties of the alloy. More specifically, it is well known that HX alloy is strengthened by the formation of carbides under specific thermal exposures, and therefore, the formed carbides must be carefully investigated [25,31]. Additionally, it is important to show a possible post-processing route to recover HX components if cracks occur.

The current work will focus on the influence of post-processing on the microstructure and hardness of LPBF HX alloy. For these reasons, a HIP treatment and solution annealing were performed on LPBF HX alloy, and the microstructures were compared to solution annealed wrought HX alloy. Finally, the post-solution annealed HX samples were subjected to thermal treatments at 745 °C for 6 hours in order to investigate the microstructure evolution simulating a thermal exposure typical for Ni-based superalloy parts [31].

## 2. Materials and Methods

**Figure 1:**
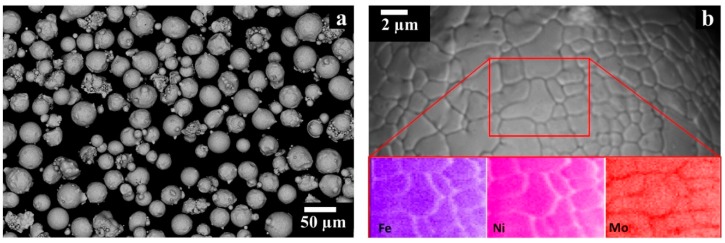
Backscattered electron (BSE) SEM images of Hastelloy X (HX) particles showing: (**a**) particle size and morphology; (**b**) dendritic microstructures of one particle with enrichment in Mo and depletion of Fe and Ni within the interdendritic areas.

The gas atomized HX powder employed in this study was supplied by LPW Technology, with a chemical composition in weight percentage (wt%) of Ni balance: Cr 21.70%, Fe 18.60%, Mo 9.20%, Co 1.82%, Si 0.36%, W 0.90%, O 0.017%, and C 0.056% [27]. The particles exhibited spherical and some irregular shapes as shown in Figure 1a, and the particle size distribution had a d10 of 24 µm and a d90 of 52 µm determined by laser granulometry (Fritsch model Analysette 22 Compact, Fritsch, Idar-Oberstein, Germany) [27]. Shells (white arrows in Figure 1a) and satellites (red arrows in Figure 1a), typical defects of the powder atomization process, are detectable though in limited amounts. The microstructure of the HX particles revealed interdendritic areas enriched in Mo caused by its high tendency to segregate, as it can be seen in Figure 1b.

Cylindrical HX specimens with a length of 77 mm and a diameter of 14 mm were fabricated along the building direction (z-axis) using an EOSINT M270 Dual Mode version (EOS GmbH, Munich, Germany) equipped with an Ytterbium 200 W fiber laser, as schematically reported in a previous work [27]. For protection of proprietary information, the process parameters are omitted.

A part of the samples was studied in the as-built state while some specimens were HIPed at 1160 °C for 4 h at 103 MPa argon pressure using standard industrial HIP parameters [32] with a slow cooling rate lower than 10 °C/min. The slow cooling rate promotes carbide formation, and therefore, another part of HIPed HX samples was subsequently solution annealed at 1175 °C (recommended temperature [25,33]) for 30 min, to dissolve the intergranular filament of carbides. The solution annealing was followed by water quenching in order to inhibit carbide re-precipitation during cooling. Different solution annealing treatments were performed at 1175 °C for 15, 30, and 60 minutes revealing that 30 min is effective in eliminating the filament of carbides.

Finally, on the post-solution annealed HX samples a heat treatment at 745 °C for 6 h followed by water quenching was performed in order to characterize the developed microstructure and the formed carbides. This temperature was selected in order to simulate a service temperature typical for Ni-based superalloy parts [31].

Table 1 reports a summary of the post-processed LPBF HX samples and corresponding treatment conditions. 

The as-built and post-processed HX specimens were cut along the building direction (z-axis) and perpendicular to the building direction (x-y plane) and then ground down to 1 micron using diamond suspension. The polished samples were analyzed by light optical microscope (LOM, Leica DMI 5000 M, Wetzlar, Germany) to observe defects such as pores and cracks, and the residual porosity of 20 LOM images taken at 100× was determined by ImageJ software. Afterwards, the samples were also etched using Kalling’s No.2 reagent (5 g CuCl_2_ in 100 mL HCl and 100 mL CH_3_CH_2_OH). The etched samples were analyzed by LOM, a scanning electron microscope (SEM, Phenom XL, Phenom-World BV, Eindhoven, The Netherlands), and a FE-SEM (field emission scanning electron microscope, Zeiss Merlin, Oberkochen, Germany) both equipped with Energy Dispersive X-ray spectrometry (EDS) to analyze the microstructure and chemical composition at different locations. By SEM and FE-SEM analyses, the microstructure was examined by backscattered electron (BSE) in order to better detect the phases by different chemical composition while secondary electron (SE) was employed to analyze their morphology at high magnification. Additionally, the grain size of post-processed HX samples was evaluated using the planimetric method on LOM micrographs according to the ASTM E112-12. 

The Rockwell B (HRB) hardness values of the as-built and post-processed HX samples were evaluated by performing five indentations on three samples for each condition using an EMCO TEST M4U test machine in accordance with the standard ASTM E18-18a.

Finally, a certain amount of carbides of the three post-processed HX samples were collected by electrolytic anodic extraction at 2 V using a solution of 25% HCl/75% CH_3_OH (in volume percentage) at room temperature, according to a method reported in a previous work by the authors for the as-built HX samples [27]. In order to study and determine the type of formed carbides within these three post-processed HX states, the extracted carbides were observed by SEM + EDS as well as characterized by X-ray diffraction (XRD, PANalytical, Almeno, The Netherland) using CuKα radiation in a Bragg Brentano configuration from 35° to 65° employing a step size of 0.013° and a counting time of 35 s per step. 

## 3. Results and Discussion 

### 3.1. Microstructure of As-Built Hastelloy X Alloy 

**Figure 2:**
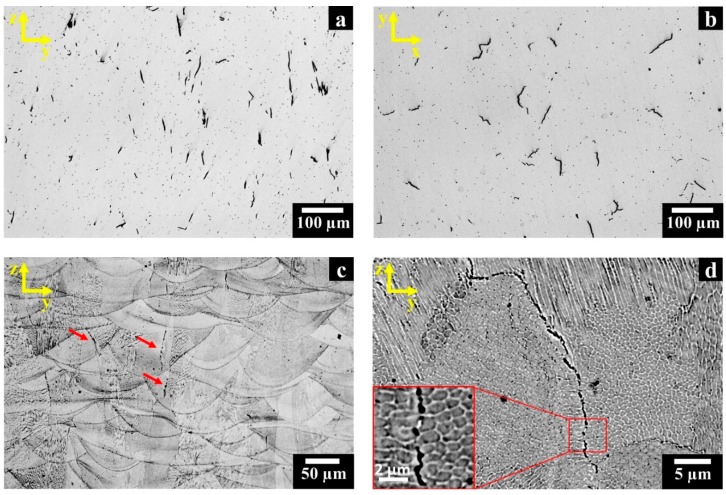
Light optical microscopy (LOM) images of as-built HX samples: (**a**,**b**) polished sample showing the cracks along the z-y and x-y planes, respectively; (**c**) etched samples revealing the microstructures and cracks along the z-y plane; (**d**) BSE SEM image of an as-built HX sample showing cellular/dendritic structures and a crack with Mo-rich carbides together with an inset of the crack.

The LOM images of polished as-built HX state reveal that cracks tend to form along the building direction (z-axis) and at random locations in the x-y plane perpendicular to the building direction, as shown in Figure 2a,b, respectively. Residual porosities of 0.29 ± 0.04% along the z-y plane and 0.34 ± 0.05% along the x-y plane were measured. The revealed pores exhibited a spherical shape, thus they probably derive from entrapped gas within the starting powder [5]. 

After etching, it is possible to observe the melt pool contours generated along the z-axis (Figure 2c) by the laser beam during the melting of powders. The heat flux dissipation along the z-axis from the top to the building platform promotes the formation of columnar grains. The cracks mainly occur along the columnar grain boundaries which represent the less resistant location path, highlighted by red arrows in Figure 2c, as well as reported in the literature for LPBF HX alloy [2,20,21,27]. Figure 2d and its inset show the presence of precipitates close to the cracks. 

In a previous work focused on the microstructure of as-built LPBF HX alloy, the authors found that the extremely fine precipitates are Mo-rich M_6_C carbides together with other possible metastable Mo-rich carbides [27]. This gives clues to the fact that Mo-rich carbides, especially those located at intergranular regions, induce material embrittlement that causes crack initiation upon the onset of the high residual stress state generated by the LPBF process. 

For the LPBF HX, the presence of very fine nanometric Mo-rich carbides was also detected in other works [23,30]. The very fine dendritic/cellular microstructures have a size typically lower than 1 µm, resulting from the melting and solidification processes which occur with segregation mechanisms acting in restricted areas and for a short time as a consequence of the high solidification rates (around 10^5^–10^6^ °C/s) [2,4,9,13,34].

### 3.2. Microstructure Evolution of Post-Processed Hastelloy X Alloy 

The HIP treatment completely consolidates the LPBF material, closing the cracks and reducing the residual porosity (less than 0.1% was recorded for both planes). Furthermore, due to the high temperature soaking and the limited heating and cooling rate, this treatment promotes recrystallization with the formation of nearly equiaxed grains along the z-axis and x-y planes.

**Figure 3:**
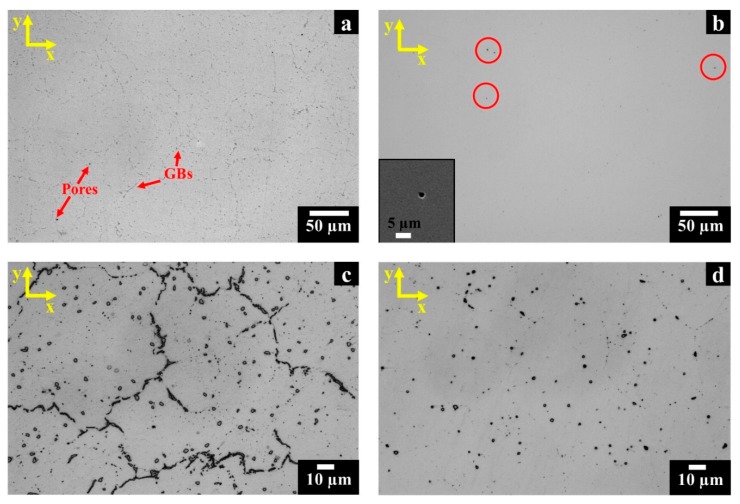
LOM images of polished post-processed HX samples along the x-y plane: (**a**) HIPed state showing pores and carbides along the grain boundaries (GBs); (**b**) post-solution annealed state exhibiting very fine isolated pores as highlighted by the SEM image in the inset; (**c**) HIPed state showing film of intergranular carbides and globular and square intragranular carbides; (**d**) post-solution annealed state exhibiting only globular and square inter/intragranular carbides.

From Figure 3a, it should be noted that several residual pores, visible as black spots, and carbides, identified by the grey spots located both along the grain boundaries and within the grains, are present in LPBF samples. Intergranular carbides formed within the HIP treatment are film-like. The analysis of the post-solution annealed HX state revealed that a dissolution of this film of carbides along grain boundaries occurred. Actually, after this treatment stage, the polished HX exhibits only very fine isolated spherical pores (as illustrated in the inset and the red circles in Figure 3b).

After etching, the optical micrographs of HIPed HX samples (Figure 3c) exhibited the inter/intragranular carbides formed during the slow cooling rate of the HIP treatment. On the other hand, after solution annealing the HX samples presented only globular and square inter/intragranular carbides (Figure 3d). For HIPed and post-solution annealed HX samples, the ASTM grain size number was assessed to be 4.5–5.5 (with grain diameters included within 53.4 and 75.5 µm), indicating that the post-solution annealing had a negligible impact on grain size. This evidences that thermal exposure at 1175 °C for 30 min is sufficient to remove the film of carbides along the grain boundaries, but the remaining intergranular carbides seem to hinder the grain growth. 

The grain size of these two post-processed states is similar to the solution annealed wrought Hastelloy X alloy reported in the literature with reference ASTM grain size number around 5 [25,26,35]. Similarly, Han et al. [29] reported recrystallization for HIPed LPBF HX samples. 

**Figure 4:**
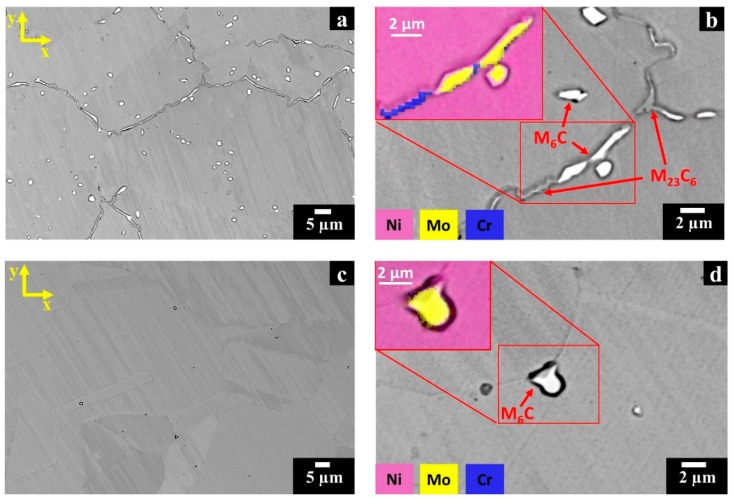
BSE SEM micrographs of post-processed HX samples along the x-y plane: (**a**,**b**) HIPed HX samples with an energy dispersive X-ray spectrometry (EDS) map showing the presence of Mo-rich M_6_C and Cr-rich M_23_C_6_ carbides; (**c**,**d**) post-solution annealed HX samples with an EDS map showing Mo-rich M_6_C carbides. Kalling’s No.2 etchant was used.

For the HIPed HX samples, the SEM + EDS investigation pointed out the formation of two different types of carbides identified as Mo-rich M_6_C and Cr-rich M_23_C_6_ carbides. In backscattered electron (BSE) mode, the Mo-rich M_6_C carbides appear bright, whereas the Cr-rich M_23_C_6_ carbides are grey as shown in Figure 4a,b.

The intragranular Mo-rich M_6_C carbides exhibit globular and square shapes with a size up to around 2.5 µm. On the other hand, elongated both Mo-rich M_6_C (up to about 15 µm) and Cr-rich M_23_C_6_ carbides (up to around 3 µm) can be observed at intergranular locations. These carbides are contiguously located along GBs so that they form a continuous film.

The SEM images of the post-solution annealed HX samples (Figure 4c,d) evidence that a complete dissolution of the elongated carbides along grain boundaries occurred, whereas that globular and square inter/intragranular Mo-rich M_6_C carbides remained unaltered maintaining dimensions similar to the HIPed condition. The current investigation highlights that the microstructures are in line with the literature on solution annealed wrought HX alloys, which consisted of austenitic (γ) matrix and loosely distributed Mo-rich M_6_C carbides throughout the material [25,26,35].

**Figure 5:**
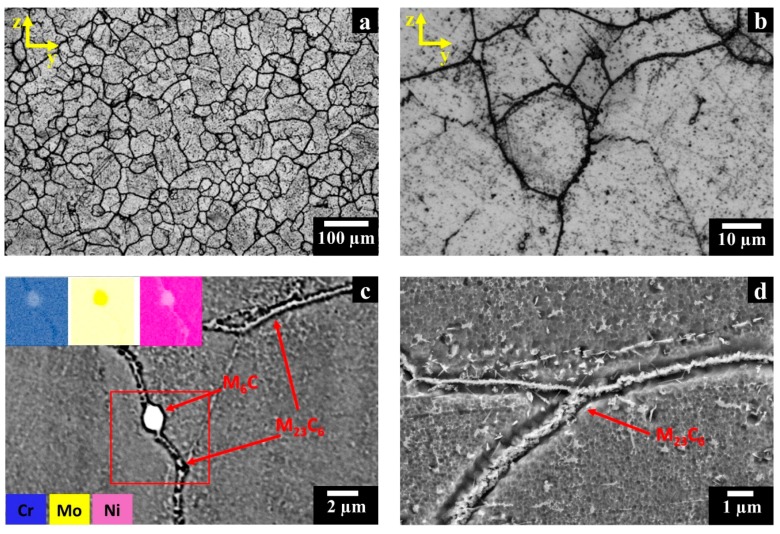
HT HX samples: (**a**,**b**) LOM images showing carbide precipitation along the grain boundaries; (**c**) BSE SEM micrograph with an EDS map showing enriched areas with Cr and Mo corresponding to Cr-rich M_23_C_6_ carbides and Mo-rich M_6_C carbides, respectively; (**d**) secondary electron (SE) FE-SEM image showing grain boundaries covered by Cr-rich M_23_C_6_ carbides. Kalling’s No.2 etchant was used.

The post-solution annealed HX samples present a high concentration of carbon within the matrix with respect to the HIPed HX state due to the dissolution of the intergranular filament carbides. Therefore, the thermal exposure at 745 °C for 6 h promoted the significant formation of carbides (Figure 5a,b), in particular filament carbides smaller than ones observed in the HIPed HX state (Figure 3c). The precipitation of intergranular carbides hinders the grain growth resulting in a grain size equal to the post-solution annealed state. 

The SEM analysis (Figure 5c) shows the presence of Cr-rich M_23_C_6_ carbides along the grain boundaries and within the grains, as also reported in the literature after treatments at around this temperature [35], as well as the presence of Mo-rich M_6_C carbides. High magnification view (Figure 5d) reveals that the intergranular Cr-rich M_23_C_6_ carbides tend to generate a continuous film exhibiting sub-micrometric thickness. 

### 3.3. Carbide Extraction

**Figure 6:**
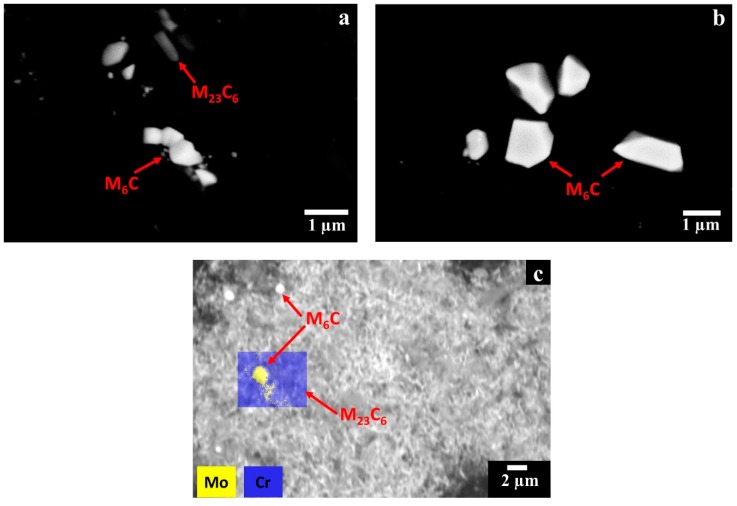
BSE SEM images of the extracted carbides within the post-processed HX states: (**a**) HIPed state showing M_6_C and M_23_C_6_ carbides; (**b**) post-solution annealed state revealing M_6_C carbides; (**c**) HT state exhibiting filaments of Cr-rich M_23_C_6_ carbides with the presence of few Mo-rich M_6_C carbides.

In order to further support carbide identification, the carbides were electrochemically extracted from the matrix. The SEM images of the extracted carbides from HIPed, post-solution annealed, and HT HX samples are provided in Figure 6a–c, whereas the EDS results of the extracted carbides are reported in Table 2. The extracted carbides of the HT HX state (Figure 6c) show a significant fraction of filament carbides identified as Cr-rich M_23_C_6_ carbides as well as a reduced number of globular and square Mo-rich M_6_C carbides.

In order to lend support to the carbide identification, the extracted carbides were also analyzed by XRD to determine their lattice parameters, evaluating the average value and standard deviation using the peaks between 35° and 65°. However, it should be noted that there are peaks of the γ phase due to the presence of matrix residuals in the extracted carbides. This was also observed by EDS analysis.

**Figure 7:**
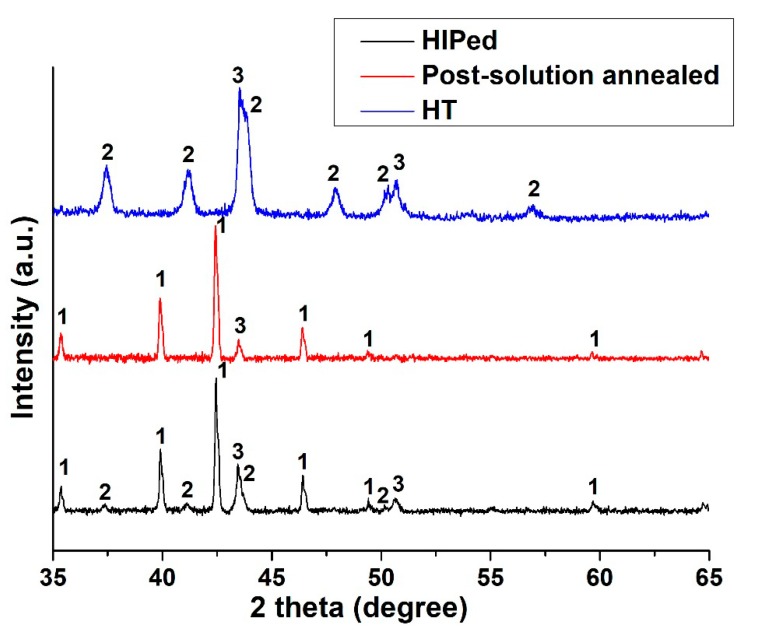
X-ray diffraction (XRD) patterns of extracted carbides from HIPed, post-solution annealed, and HT HX materials. 1 = Mo-rich M_6_C carbides, 2 = Cr-rich M_23_C_6_ carbides, 3 = γ-fcc phase.

The XRD data of the extracted carbides (Figure 7) confirmed the presence of Mo-rich M_6_C and Cr-rich M_23_C_6_ carbides with lattice parameters of 11.05 ± 0.01 Å and 10.76 ± 0.02 Å, respectively. The calculated lattice parameter of the Mo-rich M_6_C carbides is in line with the values reported in the literature for Hastelloy X alloy, which are between 10.99 Å and 11.08 Å [26,35,36]. Regarding the Cr-rich M_23_C_6_ carbides in superalloys, they have a characteristic lattice parameter from 10.50 Å to 10.70 Å [31], although an enrichment in W can increase this value [37]. 

The XRD spectrum shows a greater intensity of the Mo-rich M_6_C carbides peaks than those of Cr-rich M_23_C_6_ carbides, indicating the presence of a higher fraction of the former. This compositional feature can be explained by analyzing the time–temperature–transformation (T–T–T) diagram of the Hastelloy X alloy [36]. During cooling the formation of Mo-rich M_6_C carbides occurs both at high and low temperatures stages, whereas the formation of Cr-rich M_23_C_6_ carbides starts only at lower temperatures. Therefore, the formation of this last type of carbides is limited by the depletion of carbon already used to form the Mo-rich M_6_C carbides. For the post-solution annealed HX samples, the extracted precipitates consisted only of Mo-rich M_6_C carbides with a lattice parameter of 11.05 ± 0.01 Å. This clearly indicates that the solution annealing allows the full dissolution of Cr-rich M_23_C_6_ carbides, which then upon rapid cooling are not formed again. Thermal exposure at 745 °C for 6 h pointed out the increments of number and intensity of the peaks of Cr-rich M_23_C_6_ carbides with a lattice parameter of 10.72 ± 0.02 Å. In this state, the concentration of Cr-rich M_23_C_6_ carbides is drastically superior to the Mo-rich M_6_C carbides, as already noted in SEM investigation (Figure 6c). In this state the concentration of Mo-rich M_6_C may be under the threshold of the instrument, explaining the reason why the peaks of Mo-rich M_6_C carbides are not visible.

### 3.4. Hardness Investigation 

**Figure 8:**
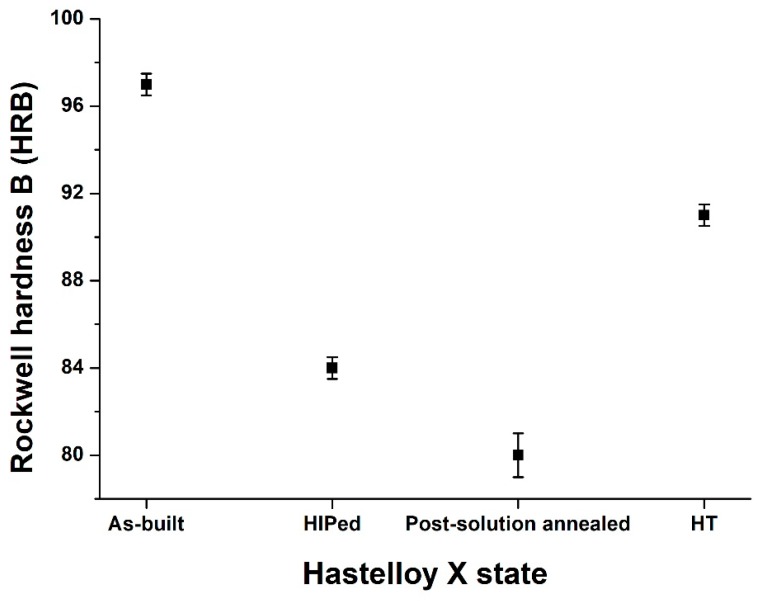
Rockwell hardness B (HRB) of as-built, HIPed, post-solution annealed, and HT HX states.

Figure 8 compares the hardness of the as-built and post-processed states. The as-built HX samples revealed a very high hardness of 97.0 ± 0.5 HRB mainly due to its very fine dendritic structures and high residual stress state. 

After HIP treatment the hardness tends to decrease to 84.0 ± 0.5 HRB due to the dissolution of the dendritic structures and residual stress relief as well as the recrystallization and grain growth. Additionally, the post-solution annealed HX state revealed a hardness of 80 ± 1 HBW, caused by carbide dissolution. In this case, the thermal treatment dissolves the intergranular film of carbides resulting in a slight hardness reduction. On the other hand, the thermal exposure at 745 °C for 6 h involves the formation of sub-micrometric carbides along the grain boundaries and within the grains leading to a drastic hardness improvement reaching 91.0 ± 0.5 HRB. 

The hardness of the commercially available solution annealed HX alloy is higher than LPBF post-solution annealed HX state with a value around 86 HRB [24], which may be caused by smaller grain size or higher concentration of fine carbides with respect to the LPBF post-solution annealed HX state.

## 4. Conclusions 

The fabrication of LPBF HX components with a complex shape can reduce the production costs compared to traditional processes. Nevertheless, post-processing treatments are crucial to generate a tailored microstructure for high-temperature applications.

In this work, it is demonstrated that a standard HIP treatment could close the internal cracks, reducing the residual porosity (residual porosity less than 0.1%) as well as generating equiaxed grains along x-y and z-plane. However, the typical slow HIP cooling rate involved the formation of globular and square Mo-rich M_6_C carbides mainly within the grains and elongated mixed Mo-rich M_6_C and Cr-rich M_23_C_6_ carbides along the grain boundaries, thus forming a continuous film. A subsequent solution annealing was then performed, resulting in the dissolution of the film of carbides at grain boundaries. On the other hand, globular and square Mo-rich M_6_C carbides remained practically unaltered. Furthermore, it is observed that the post-solution annealed HX samples consisted of equiaxed grains similar to solution annealed wrought HX alloy. Finally, simulating a thermal exposure compatible with components made of Ni-based superalloys (745 °C for 6 h), the HT HX samples revealed the formation of sub-micrometric Cr-rich M_23_C_6_ carbides along the grains boundaries as well as inside the grains, presenting a microstructure evolution similar to traditional annealed wrought HX alloy. 

The current microstructural investigation was performed on LPBF HX with a standard chemical composition. The use of modified HX powder, in particular, HX powder with a reduced quantity of carbon can reduce or inhibit the crack formation but limits the carbide precipitation under post thermal treatments or service at high temperatures. The carbides play a primary role in the strengthening of the HX alloy, and consequently, a lower formation of carbides can have an impact on the mechanical properties. Therefore, future studies should take into account the mechanical properties of LPBF HX alloy with standard and modified chemical composition under thermal exposures.

The main findings of the current work highlight the possibility of using a post-processing route to obtain post-solution annealed LPBF HX with an extremely high densification level (residual porosity <0.1%) as well as to generate tailored microstructure features in line with the traditional solution annealed wrought HX alloy and, consequently, suitable for high-temperature applications. In addition, for the post heat-treated LPBF HX alloy, the investigation points out the significant carbide formation and their strengthened effect.

## Figures and Tables

**Table 1:** Three post-processed conditions investigated: hot isostatic pressed (HIPed), post-solution annealed, and heat-treated (HT).

State	HIPed	Post-Solution Annealed	HT
**Heat treatment**	1160 °C 4 h 103 MPa	1175 °C for 30 min	745 °C for 6 h
**Cooling rate**	<10 °C/min	Water quenched	Water quenched

**Table 2:** EDS results in weight percentage of the extracted carbides from HIPed, post-solution annealed, and HT HX samples.

HX State	Carbide	Ni	Cr	Fe	Mo	Co	W	Si
**HIPed**	Mo-rich M_6_C	14.7	12.3	4.6	53.6	-	11.4	3.4
	Cr-rich M_23_C_6_	4.8	63.1	4.9	21.0	-	4.8	1.4
**Post-solution annealed**	Mo-rich M_6_C	13.6	12.3	5.2	51.8	0.7	13.4	3.0
**HT**	Cr-rich M_23_C_6_	9.1	56.3	6.3	21.9	0.3	4.2	1.9
